# Bioinformatics analysis of the mechanisms and efficacy of the Bushen Anzhi recipe in treating aging-related insomnia

**DOI:** 10.3389/fpsyt.2026.1770410

**Published:** 2026-05-08

**Authors:** Haiming Li, Ruining Liang, Zhenhui Li, Wenjun Cui, Yanling Hu, Guangke Zuo, Hailong Zhu, Xingping Zhang

**Affiliations:** 1 The Fourth Clinical Medical College of Xinjiang Medical University, Urumqi, China; 2 College of Traditional Chinese Medicine, Xinjiang Medical University, Urumqi, China; 3 Affiliated Hospital of Traditional Chinese Medicine of Xinjiang Medical University, Urumqi, China

**Keywords:** aging-related insomnia, biological mechanism, Chinese herbal medicine, EEG monitoring, network pharmacology

## Abstract

**Background:**

Chinese herbal medicine has a long history of treating insomnia with excellent curative effects; however, the underlying mechanisms remain unclear. This study discusses the mechanism and curative effects of the main Chinese herbal medicines in the Bushen Anzhi recipe for managing aging-related insomnia (ARI).

**Methods:**

The insomnia dataset GSE98582 was obtained from the Gene Expression Omnibus database and included 342 samples from individuals with insomnia and 213 control samples. In total, 931 neuroinflammatory targets were identified in the GeneCards and molecular signatures (MSigDB) databases. The active components corresponding to *Radix Rehmanniae Preparata*, *Cornus officinalis*, *Rhizoma dioscoreae*, *Poria cum Radix Pini*, and *Polygala tenuifolia* were obtained from the Traditional Chinese Medicine Systems Pharmacology and HERB databases. Multi-level interactive network and enrichment analyses of overlapping targets were performed using the STRING, MSigDB, and GeneMANIA websites. Sixty male Sprague Dawley rats were divided into three groups: control (Con, n=20), aging-related insomnia (ARI, n=20), and aging-related insomnia Bushen Anzhi recipe treatment (ARI-BSAZ, n=20). In the ARI group, d-galactose and parachlorophenylalanine were injected intraperitoneally to create an aging-related insomnia rat model. Bushen Anzhi formulation was administered by gavage; Polysomnography, Morris water maze, open-field tests, western blot, and ELISA analyses were performed to assess the effects of this Chinese herbal medicine in managing insomnia.

**Results:**

A total of 25 effective components and 16 neuroinflammation and drug target-related differentially expressed genes were screened. Multi-level network and enrichment analyses showed that Bushen Anzhi mainly regulated cellular biological processes through core target genes such as HIF1A, PTGS2, and GSK3B. Furthermore, compared with the ARI group, the Bushen Anzhi recipe was also found to contribute to increases in total sleep and slow-wave sleep times; significant reductions in escape latency time; increases in fourth quadrant residence time, average movement speed, and amount of wall climbing; reductions in the levels of GSK-3β; and increases in the levels of β-catenin and Wnt3a.

**Conclusion:**

The Bushen Anzhi recipe may alleviate ARI through a complex multi-level interaction network, with findings suggesting a potential association with the regulation of the Wnt signaling pathway, though further experimental validation is warranted.

## 1 Introduction

Insomnia is common in clinical practice, occurring in as many as 50% of outpatients, and significantly affects patients’ quality of life as well as broader societal and economic outcomes. It is also a risk factor for the development and deterioration of other diseases (1). Disruptions in sleep quality and continuity may aggravate psychological symptoms in patients with insomnia, and these symptoms may in turn exacerbate insomnia severity (2). In older adults, age-related decreases in upper airway muscle tension lead to an increased incidence of obstructive sleep apnea (3). Several neurodegenerative diseases, such as dementia and Parkinson’s disease, disrupt neural network structures (basal forebrain and brain stem nucleus) in sleep–wake regulation, leading to circadian rhythm disorders (4). In addition, dysfunction of the suprachiasmatic nucleus in older individuals also leads to sleep fragmentation and early awakening (5).

Currently, the treatment of insomnia includes drug therapy and cognitive behavioral therapy; however, these approaches have limitations, including moderate therapeutic efficacy and potential adverse effects (6, 7). Traditional Chinese medicine has extensive clinical use and includes herbal prescriptions, acupuncture, and massage. The efficacy of Chinese herbal medicine in managing insomnia is supported by clinical evidence (8, 9). Traditional Chinese medicine may alleviate aging-related insomnia (ARI) symptoms by regulating the gut microbiota (10). For example, center-supplementing and qi-boosting decoction and spleen-tonifying and yin heat-clearing decoction were reported to significantly improve sleep quality within two weeks. The intestinal microflora and inflammatory markers also significantly improved after treatment (11).

Professor Zhang Xingping has used the Bushen Anzhi recipe to treat insomnia in older adults for several years, achieving excellent therapeutic effects. The commonly used herbal components in this recipe are *Radix Rehmanniae Preparata* (RRP; Shudihuang), *Cornus officinalis* (CO; Shanzhuyu), *Rhizoma dioscoreae* (RD; Shanyao), *Poria cum Radix Pini* (PCRP; Fushen), and *Polygala tenuifolia* (PT; Yuanzhi). RRP can improve cognitive dysfunction and neuronal pathological changes in Alzheimer’s disease model mice by regulating AKT/GSK-3β signaling pathway and the gut microbiota (12). Loganin, the main extract of *Cornus officinalis*, increases the expression of c-Fos in GABAergic neurons in the ventrolateral preoptic nucleus of rats and decreases the levels of 5-hydroxytryptamine (5-HT), dopamine (DA), and 3,4 dihydroxyphenylacetic acid in the prefrontal cortex, resulting in beneficial sedative and hypnotic effects (13). *Rhizoma dioscoreae* extracts (e.g., dioscin and yam polysaccharides) can reduce the levels of inflammatory markers in the brain (14). *Poria* contains triterpenoids and polysaccharides, which have anti-inflammatory and mood-stabilizing effects and can influence hippocampal neuronal development in neonatal rats through the Rho signaling pathway (15). The sedative and hypnotic effects of PT on rats with aging-related insomnia may be related to neuronal and metabolic pathways, especially the gamma-aminobutyric acid (GABA) energy signaling pathway (16). Polygalanin extract has neuroprotective effects in various nervous system diseases, and its mechanisms include promoting autophagy, inhibiting apoptosis, and reducing oxidant stress associated with misfolded proteins (17).

The mechanism of action of traditional Chinese medicine prescriptions involves multiple targets, and this multi-target nature aligns with integrative and systems-level framework of network pharmacology; thus, network pharmacology is suitable for studying the pharmacological mechanisms of traditional Chinese medicine compounds (18). The sedative and sleep-promoting properties of Chinese herbal medicines depend on their interactions with various neurotransmitter systems in the brain, among which 5-HT plays an important role in insomnia (19). Chinese herbal medicines have the potential to improve sleep quality; however, the specific mechanism remains unclear. Traditional Chinese Medicine Network Pharmacology (TCM-NP) can be used to predict the active components of traditional Chinese medicine and their corresponding protein targets according to the literature in various databases and reveal the therapeutic mechanism of Chinese herbal medicine for complex diseases from massive omics data (20).

In this study, we aimed to systematically mine biological information in large-scale data and construct the disease–traditional Chinese medicine-component–target interaction network using dataset correction, differential expression analysis, enrichment analysis, and protein-protein interaction network construction, to reveal the potential relationship between the active components of traditional Chinese medicine and insomnia-related genes. We also aimed to assess the *in vivo* efficacy of traditional Chinese medicine prescriptions of RRP, CO, RD, PCRP, and PT in Sprague Dawley (SD) rats and to confirm the efficacy of the Bushen Anzhi recipe in treating ARI by monitoring electroencephalograms (EEG) and electromyograms (EMG), thus providing a theoretical basis for the clinical treatment of older adults with insomnia.

## 2 Materials and methods

### 2.1 Data download

The insomnia dataset GSE98582 was downloaded from the Gene Expression Omnibus (GEO) database (https://www.ncbi.nlm.nih.gov/geo/) using the R package GEOquery (Version 2.76.0). The samples were from *Homo sapiens*, and the tissue source was blood from 342 individuals with insomnia and 213 control samples (**Table 1**). We identified 929 neuroinflammation-related genes (NRGs) in the GeneCards database (https://www.genecards.org). In total, 166 NRGs were obtained from the molecular signatures database (MSigDB), and 931 NRGs were obtained after merging (**Supplementary Table 1**).

**Table 1:** GEO microarray chip information.

Characteristics	GSE98582
Platform	GPL6244
Species	*Homo sapiens*
Tissue	blood
Samples in insomnia group	342
Samples in control group	213
Reference	PMID:29739334

GEO, Gene expression omnibus.

### 2.2 Drug component screening and target protein prediction

Using absorption, distribution, metabolism, and excretion data in Traditional Chinese Medicine Systems Pharmacology (TCMSP) (https://www.tcmsp-e.com/tcmspsearch.php) database, according to the oral availability > 30% and drug-likeness > 0.18, the predicted protein targets of active components of Shudihuang, Shanzhuyu, and Shanyao were screened (**Supplementary Table 2**), and the predicted targets screened in TCMSP were transformed into gene names by UniprotKB (http://www.uniprot.org). According to the criterion *p* < 0.05, the targets corresponding to Yuanzhi and Fushen were obtained from the HERB database (http://herb.ac.cn/). Subsequently, 244 drug target-related genes (DTRGs; **Supplementary Table 3**) were obtained by merging and summarizing the above databases, and the component–target network of traditional Chinese component–targets was visualized using Cytoscape software.

### 2.3 Insomnia-related differentially expressed genes

The intersection of NRGs and DTRGs was taken, and a Venn diagram was drawn to obtain neuroinflammation and drug target-related genes (NDTRGs). Differential expression analysis was conducted on dataset GSE98582 to compare gene expression profiles between the insomnia group and the control group using the R-package limma (Version 3.64.1). The raw expression matrix was processed by mapping probes to their corresponding genes based on the official annotation file of platform GPL6244, with expression values of duplicate probes consolidated through summarization. Subsequently, the expression data were normalized using the limma package to enhance inter-sample comparability. All *p*-values were corrected using the Benjamini–Hochberg method (21). During the differential expression analysis, a linear model was constructed using lmFit(), inter-group contrasts were specified via makeContrasts(), the model was refitted using contrasts.fit(), and empirical Bayes moderation was applied through eBayes() to derive differential expression statistics. Differentially expressed genes (DEGs) were defined using thresholds of |logFC| > 0.05 and *p* < 0.05. Insomnia-associated transcriptional alterations typically manifest as pervasive, low-amplitude yet highly coordinated transcriptional reprogramming, rather than large-magnitude expression fluctuations confined to a limited number of genes (22). The application of an excessively stringent logFC threshold would lead to the systematic exclusion of such genes, thereby substantially undermining the comprehensive elucidation of the molecular mechanisms underlying insomnia (23). The selected threshold was designed to balance sensitivity and specificity in order to capture biologically meaningful signals. Downstream analyses, including Gene Ontology enrichment, Kyoto Encyclopedia of Genes and Genomes pathway analysis, Gene Set Enrichment Analysis, and Gene Set Variation Analysis, were subsequently employed to mitigate analytical bias (**Supplementary Figure 1**). The volcano plot was drawn by ggplot2 (Version 3.5.2) of the R package, intersected with NDTRGs, and the Venn diagram was drawn to obtain neuroinflammation and drug target-related differentially expressed genes (NDTRDEGs). The heatmap was drawn by pheatmap (Version 1.0.13) of the R package.

### 2.4 Function and pathway enrichment analysis

Gene Ontology (GO) analysis is a method used for large-scale functional enrichment research, including biological processes (BP), cellular components (CC), and molecular functions (MF). The Kyoto Encyclopedia of Genes and Genomes (KEGG) database contains information on genomes, biological pathways, diseases, and drugs. We used the R-package clusterProfiler (Version 4.16.0) to analyze the GO and KEGG enrichment of NDTRDEGs, and the screening criteria were *p* < 0.05 and false discovery rate (FDR, *q*) < 0.05.

### 2.5 Gene set enrichment analysis

Gene set enrichment analysis (GSEA) was used to evaluate the distribution trend of gene-phenotype correlation rankings to determine their contribution to the phenotype. The genes of GSE98582 were sorted according to the logFC value between the insomnia and control groups, and then the genes were subjected to GSEA using the R-package clusterProfiler (Version 4.16.0). The parameters were as follows: the seed was 2020, and each gene set included at least 10 genes and at most 500 genes. Gene set c2.all.v2023.2.Hs.symbols was obtained from the MSigDB for GSEA, and the screening criteria were *p* < 0.05, and FDR (*q*) < 0.05.

### 2.6 Gene set variation analysis

Gene Set Variation Analysis (GSVA) is a nonparametric unsupervised analysis method, which is mainly used to evaluate whether different pathways are enriched among different samples. The c2.cp.v2023.2.Hs.symbols.gmt gene set was obtained from the MSigDB database, and all genes in the dataset GSE98582 were analyzed using the R-package GSVA (Version 2.2.0). The differences in functional enrichment between the insomnia and control groups were calculated. The screening standard was *p* < 0.05.

### 2.7 Protein-protein interaction

The protein-protein interaction (PPI) network was constructed by using the STRING database (Version 12.0) to set the biological species as human, and a medium confidence level ≥ 0.4, and the PPI network model was visualized by Cytoscape. Using five algorithms of degree correlation, maximum neighborhood component, maximum clique centrality, edge percolated component, and density of maximum neighborhood component in the cytoHubba plug-in, the top six shared NDTRDEGs were selected as key genes. We used the GeneMANIA website to predict functionally similar genes among the selected key genes and build an interaction network.

### 2.8 Expression difference analysis, correlation analysis, and receiver operating characteristic curve analysis

The Spearman algorithm was used to analyze the correlation between the expression levels of key genes, and the results were displayed by drawing a correlation heat map with the R-package pheatmap (Version 1.0.13). The correlation was deemed to be weak or uncorrelated if the absolute value of the correlation coefficient (r value) was below 0.3, weak if it was between 0.3 and 0.5, moderate between 0.5 and 0.8, and strong if above 0.8.

### 2.9 Functional similarity analysis

Semantic comparison of GO tags provides a quantitative method for similarity calculation between genes and genomes and has become an important basis for many bioinformatics analysis methods. The GO semantic similarity of key genes was calculated using the GOSemSim package (Version 2.34.0), and the geometric averages of key genes at BP, CC, and MF levels were calculated. Finally, the results of the functional similarity analysis were visually displayed using the ggplot package.

### 2.10 Animal grouping and Chinese medicine intervention

Sixty specific pathogen-free (SPF) Sprague–Dawley rats, six weeks old, weighing between 220 and 230 g, were purchased from Xinjiang Medical University (production license number: SCXK 2023-0001), and randomly assigned to one of the following three groups: control (Con; n = 20), aging-related insomnia model group (ARI; n = 20), and aging-related insomnia treated with the Bushen Anzhi recipe (ARI-BSAZ; n = 20). The control group did not receive any treatment; the insomnia model group was given D-galactose 120 mg/kg/d subcutaneously in the back of the neck for 42 days, then PCPA 300 mg/kg/d intraperitoneally for three days (24). The combination of D-gal (to induce the aging-like state) and PCPA (to disrupt serotonin-dependent sleep regulation) was designed to recapitulate the key neuroimmune and sleep-regulatory features of ARI in a controlled and reproducible experimental system (25, 26). Meanwhile, we monitored the EEG of the control group and the insomnia model group to make sure that the model was made successfully (**Supplementary Figure 2**). Bushen Anzhi recipe consists of Shudihuang 15 g, Shanzhuyu 20 g, Shanyao 15 g, Fushen 10 g, and Yuanzhi 12 g. It was decocted in 500 mL of water for 1 h and given to the rats by gavage at a dose of 6.4 g/kg/d (The dose is based on the **Supplementary Material**, **Supplementary Figure 3**). This study was approved by the Experimental Animal Ethics Committee of Xinjiang Medical University (No: IACIC-JT-20250527-07).

### 2.11 Sleep monitoring by polysomnography

After isoflurane anesthesia, hair was removed from the scalp of the rats in each group. After iodophor disinfection, an incision of approximately 1.5 cm was cut along the sagittal suture to expose the skull. A small hole with a diameter of 1 mm was drilled using a miniature drill, 2 mm in front of the coronal suture, 4 mm lateral at the intersection of the left and right sides of the sagittal suture, 2 mm at the intersection of the herringbone suture, and 4 mm at the left and right sides of the sagittal suture. The electrodes were connected to the corresponding leads of EEG1, EEG2, and EMG, with two electrodes used for each group, and fixed with dental cement (**Supplementary Figure 4**). Sleep cycles were automatically recorded and analyzed using the Sirenia^®^ Sleep Pro system (Pinnacle Technology, Lawrence, KS, USA). The agreement between automated and manual scoring was assessed using Cohen’s kappa coefficient, with kappa values of ≥ 0.80 achieved across all animals, confirming the reliability of the automated scoring system. Epochs with ambiguous or discordant stage classifications were jointly reviewed by three independent scorers, and a consensus decision was adopted as the definitive staging outcome. The rats were allocated to different groups based on the use of a random number table. They were maintained at a temperature of 24 ± 1 °C and relative humidity of 50 ± 5% under a 12h-light/12h-dark photoperiod, adapting well to this environment and eating freely. The epoch duration was set to ten seconds, recording was continued for one week, the signal noise and artificially affected data were deleted. Criteria for Sleep Stage Scoring (27, 28). (1) Wake: Characterized by high-frequency, low-amplitude mixed EEG activity (predominantly β-waves, >13 Hz) accompanied by high electromyographic (EMG) tone. (2) NREM sleep: Defined by high-amplitude, low-frequency EEG activity dominated by delta waves (0.5–4 Hz, comprising >20% of total EEG power), with reduced but present EMG tone. (3) REM sleep: Identified by low-amplitude, mixed-frequency EEG with a prominent theta rhythm (5–9 Hz), accompanied by near-complete EMG atonia.

### 2.12 Behavioral test

Ten rats in each group were subjected to the Morris water maze (MWM) test (WMT-200, Chengdu Techman, China), and the time required for the rats to find the platform within 60 s was recorded; this was repeated for 6 d. On the 8th day, space exploration test was carried out, escape latency time, times of crossing the platform, and swimming time in the platform quadrant were recorded within 120 s. The open field test (OFT) (JLBehv-LAR, Shanghai Jiliang, China) was performed in an area with a dimension of 81 cm × 81 cm × 28 cm. Rats were placed facing the wall and allowed to explore the environment freely for 5 min. Each rat was tested three times, and the average speed and time of climbing the wall were calculated.

### 2.13 WB and ELISA detection

After behavioral test, ten rats were randomly selected from each group (30 rats in total), rat hippocampus tissue was homogenized with lysis solution (containing 1% PMSF) to extract protein, and the protein concentration was detected by BCA kit (PC0020, Solarbio, China). Samples were added to a pre-cast polyacrylamide gel, electrophoresed in a Running Buffer containing 10% SDS, transferred to PVDF membrane, and Anti-Wnt3a (1: 800, GB153750, Servicebio, China), Anti-β-catenin(1:1000, GB150016, Servicebio, China), Anti-β-tubulin(1:1000, GB15140, Servicebio, China) were incubated overnight, and the secondary antibody was added, and the automatic luminometer was used. According to the instruction of ELISA kit, the content of GSK-3β(ER0060, FineTest, China) in tissue supernatant was detected by enzyme-labeled instrument (VICTOR Nivo, Perkin Elmer, USA).

### 2.14 Statistical analysis

Data analysis was performed using R software (Version 4.2.2). To compare two groups of continuous variables, the statistical significance of normally distributed variables was estimated using the independent Student’s *t*-test, and differences between non-normally distributed variables were analyzed using the Mann-Whitney U-test. The Kruskal-Wallis test was used to compare three or more groups. The correlation coefficients of different molecules were calculated using Spearman correlation analysis, and *p* < 0.05 was considered statistically significant. In functional enrichment analyses (including GO, KEGG, GSEA, and GSVA), the false discovery rate (FDR) was corrected for multiple comparisons, whereas the results of other comparisons based on the original *p* values were mainly used for the purposes of exploratory analysis, and their statistical significance should be interpreted with respect to the biological background (details are in **Supplementary Table 4**).

## 3 Results

### 3.1 Data set correction

**Figure 1:**
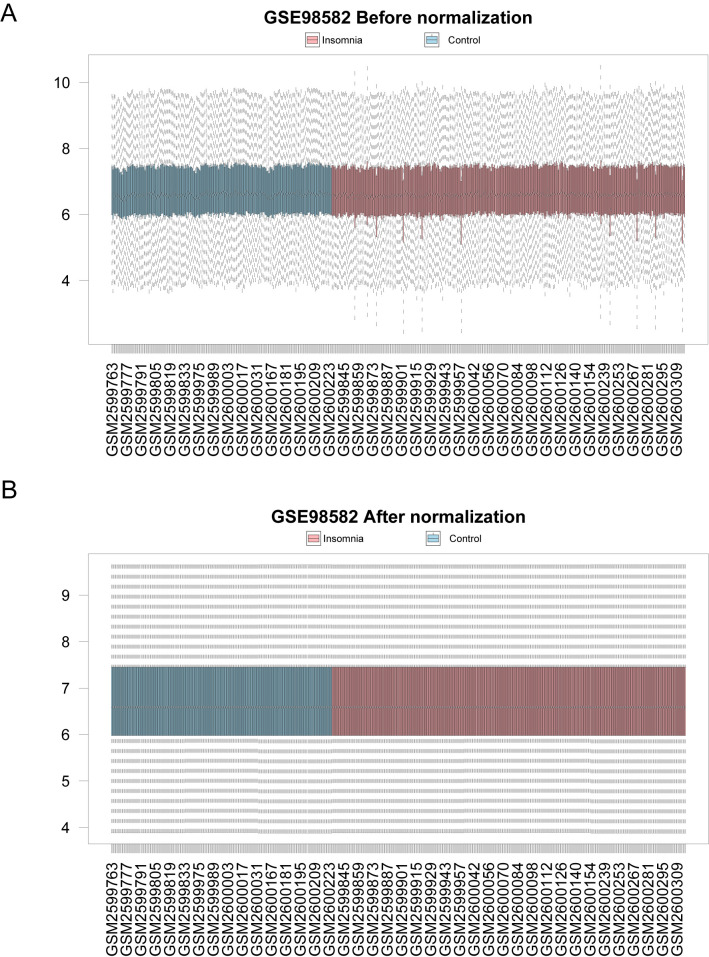
Before and after dataset normalization. **(A)** Distribution boxplot of gene expression between GSE98582 samples before correction. **(B)** Corrected distribution box diagram of gene expression between GSE98582 samples. Pink represents the insomnia group, and blue represents the control group.

The GSE98582 dataset was divided into the insomnia and control groups, data cleaning operations such as standardization and annotation probes were carried out, and a boxplot of the data distribution before and after standardization was drawn. After standardization, the expression levels of different samples in the dataset tended to be consistent (**Figures 1A, B**).

### 3.2 Screening of active ingredients and insomnia-related targets in Bushen Anzhi recipe

**Figure 2:**
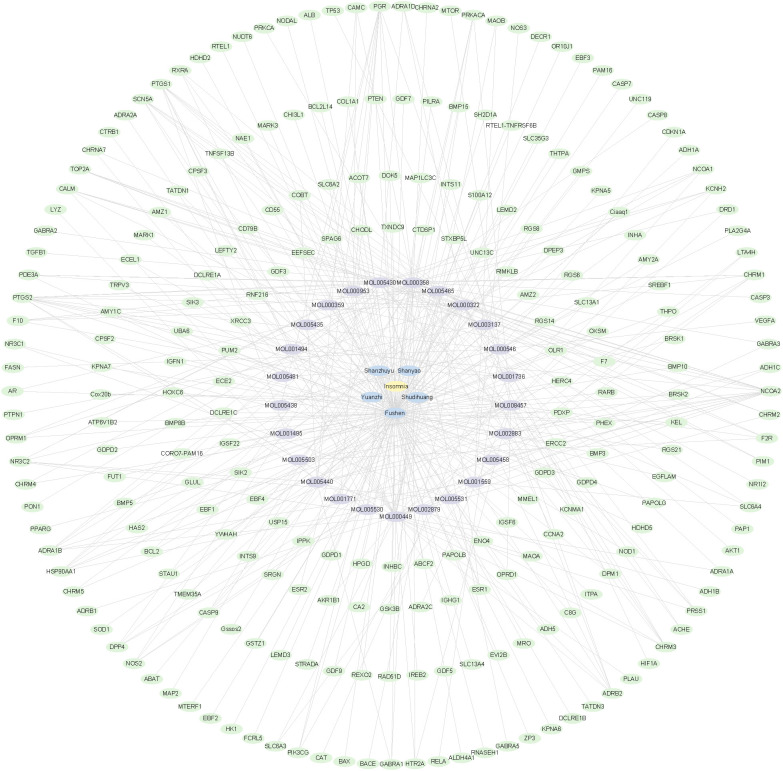
Disease–traditional Chinese medicine ingredient–target interaction network. A. Disease–traditional Chinese Medicine ingredient–target interaction network. The yellow oval node represents the name of the disease. Blue oval nodes represent five traditional Chinese medicines, purple oval nodes represent bioactive compounds in medicines, and green oval nodes represent target genes in insomnia.

Information on the active ingredients of the drugs was obtained from the database (**Supplementary Table 2**), including 14 compounds from Shanzhuyu, 12 from Shanyao, and 2 from Shudihuang (**Supplementary Table 5**). The TCMSP database was used to predict the protein targets corresponding to the active ingredients. UniProtKB was used to convert them into gene names, and 96 active ingredient targets of the Chinese herbal compounds were obtained (**Supplementary Table 6**). Targets of Yuanzhi (n=4) and of Fushen (n=146) were obtained from the HERB database (**Supplementary Tables 7**, **8**), and the drug targets were used to construct a disease–herbal medicine–ingredient–target interaction network (**Figure 2**).

### 3.3 Differentially expressed genes in drug targets related to insomnia

**Figure 3:**
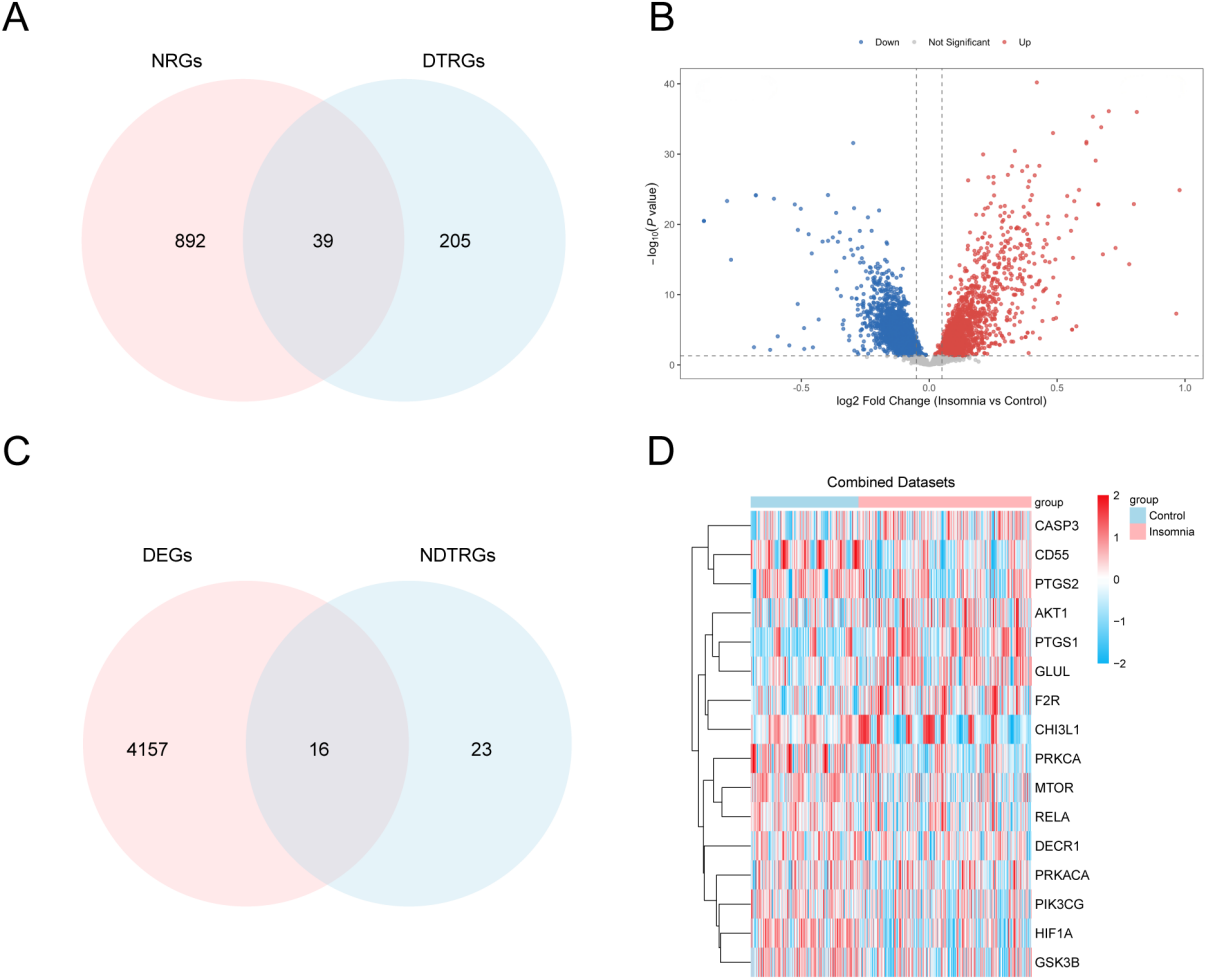
Differential gene expression analysis. **(A)** Venn diagram of NRGs and DTRGs. **(B)** Volcano plot of differentially expressed genes between the insomnia group and the control group in data set GSE98582. **(C)** Venn diagram of DEGs and NDTRGs in data set GSE98582. **(D)** Heatmap of NDTRDEGs in data set GSE98582. NRGs, neuroinflammation-related genes; DTRGs, drug target-related genes; DEGs, differentially expressed genes; NDTRDEGs, neuroinflammation and drug target-related differentially expressed genes. Pink is the insomnia group, and blue is the control group. In the heatmap, red represents high expression and blue represents low expression.

To obtain the NDTRDEGs associated with insomnia, we crossed 931 NRGs and 244 DTRGs and drew a Venn diagram (**Figure 3A**) to obtain 39 NDTRGs (**Supplementary Table 9**). To investigate differential gene expression between the insomnia group and the control group in dataset GSE98582, differential expression analysis was performed using the R package limma. A total of 4,173 differentially expressed genes (DEGs) were identified based on the threshold criteria of |log FC| > 0.05 and *p* < 0.05. Among these, 1,505 genes were significantly upregulated (log FC > 0.05 and *p* < 0.05), while 2,668 genes were significantly downregulated (log FC < −0.05 and *p* < 0.05). The results of the differential expression analysis are presented in the volcano plot (**Figure 3B**; **Supplementary Table 10**).The DEGs and NDTRGs were intersected, and the Venn diagram was drawn (**Figure 3C**). Sixteen NDTRDEGs were identified: prostaglandin-endoperoxide synthase 1 (PTGS1), PTGS2, decay-accelerating factor (CD55), protein kinase cα (PRKCA), hypoxia inducible factor-1α (HIF1A), F2R, GSK3B, CASP3, MTOR, GLUL, RELA, CHI3L1, AKT1, PIK3CG, DECR1, and PRKACA. Based on the intersection results, a heatmap was drawn using the R-package pheatmap to depict the analysis results of the 16 NDTRDEGs (**Figure 3D**).

### 3.4 Gene ontology and pathway enrichment analysis

**Figure 4:**
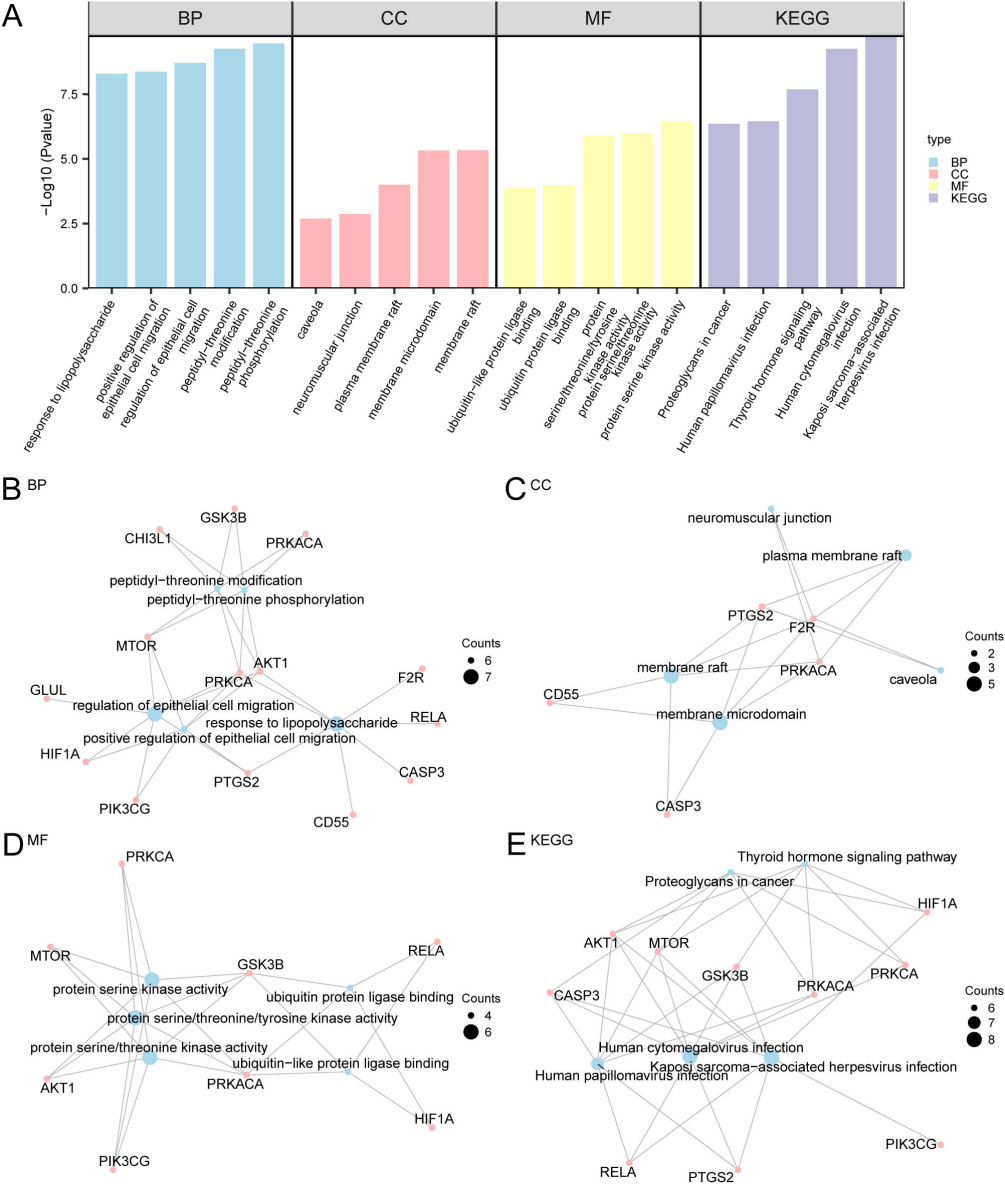
GO and KEGG enrichment analysis for NDTRDEGs. **(A)** Histogram enrichment analysis results of GO and KEGG of NDTRDEGs shows: BP, CC, MF, and KEGG. The abscissa represents GO terms and KEGG terms. **(B-E)** Network diagram of enrichment analysis results of GO and KEGG of NDTRDEGs: BP **(B)**, CC **(C)**, MF **(D)** and KEGG **(E)**. Blue nodes represent items, pink nodes represent molecules, and connecting lines represent the relationship between items and molecules. GO, gene ontology; KEGG, Kyoto encyclopedia of genes and genomes; BP, biological process; CC, cellular component; MF, molecular function. The screening criteria for enrichment analysis of GO and KEGG are *p* < 0.05 and FDR (*q*)< 0.05.

Gene Ontology (GO) and pathway (KEGG) were enriched in these 16 NDTRDEGs. The results showed that NDTRDEGs were mainly involved in BP such as peptide-tyrosine phosphorylation and peptidyl-threonine modification in insomnia; CC such as membrane raft, membrane microdomain, and neuromuscular junction; and MF such as protein serine/threonine kinase activity and others. It was also enriched in biological pathways, such as the thyroid hormone signaling pathway (KEGG) (**Figure 4A**). Simultaneously, according to the GO and KEGG enrichment analyses, a network diagram (**Figures 4B–E**) was drawn. The network diagram shows relationships between the NDTRDEGs and the corresponding entries. The larger the node is, the more molecules the entry contains.

### 3.5 Gene set enrichment analysis

**Figure 5:**
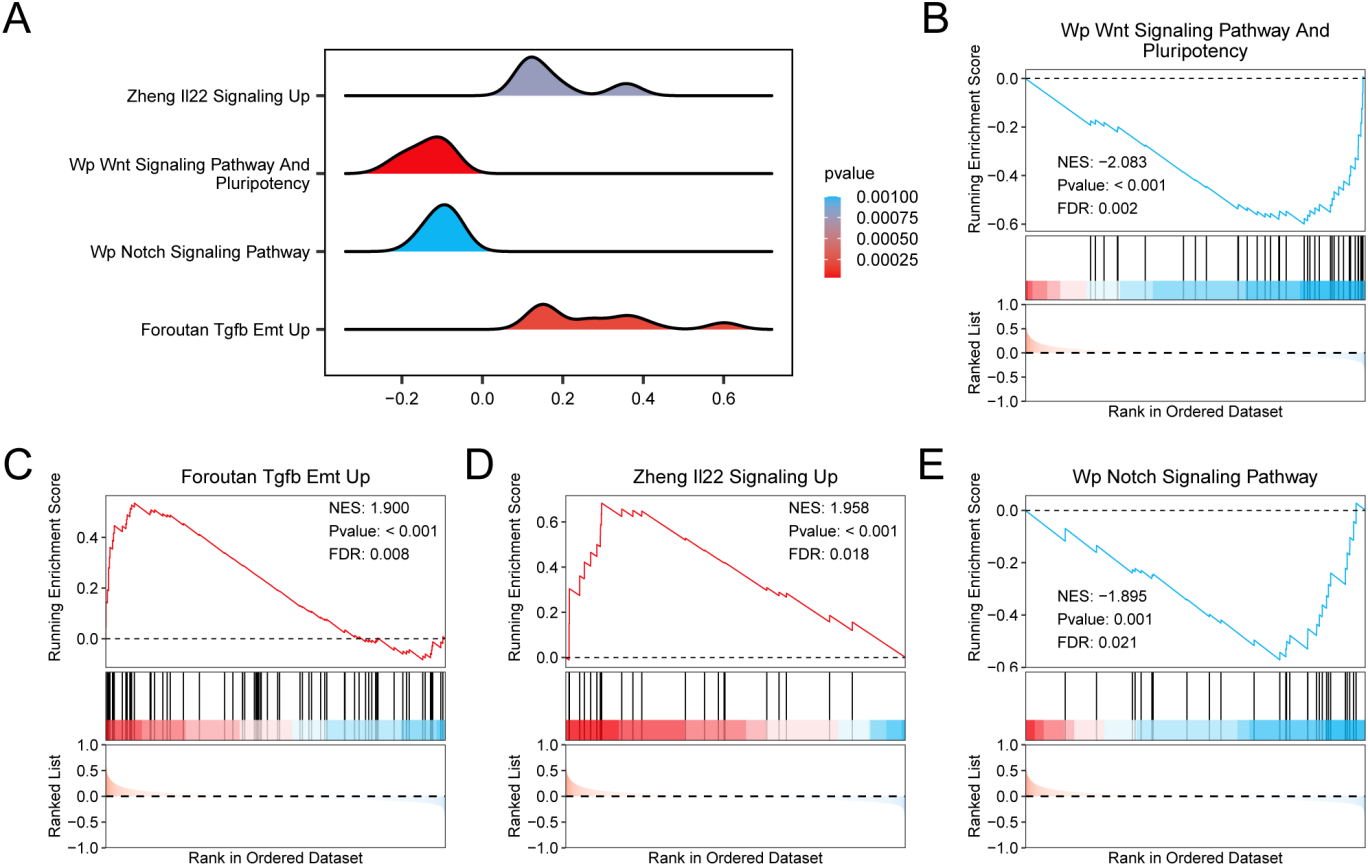
Differential gene expression analysis and GSEA for GSE98582. **(A)** Enrichment curve of the four biological functions of GSEA in data set GSE98582 is displayed. **(B-E)** The data set is significantly enriched in Wp Wnt Signaling Pathway And Pluripotency **(B)**, Foroutan Tgfb Emt Up **(C)**, Zheng Il22 Signaling Up **(D)**, Wp Notch Signaling Pathway **(E)**. GSEA, gene set enrichment analysis. GSEA screening criteria are *p* < 0.05 and FDR value (*q*)< 0.05.

To determine the influence of the expression level of all genes in the GSE98582 dataset on the onset of insomnia, gene expression and the biological processes involved were studied by GSEA and displayed on a ridge plot (**Figure 5A**). The specific results are presented in **Table 2**. All genes were significantly enriched in the Wnt signaling pathway, pluripotency (**Figure 5B**), Foroutan Tgfb Emt Up (**Figure 5C**), Zheng Il22 signaling Up (**Figure 5D**), Notch signaling pathway (**Figure 5E**), and other biological functions and signaling pathways.

**Table 2:** Results of GSEA for GSE98582.

ID	Set Size	Enrichment score	NES	*P-*value	p. adjust	*q-*value
Wp Wnt Signaling Pathway And Pluripotency	37	-0.600254567	-2.083226648	3.49E-05	0.002645543	0.002216687
Foroutan Tgfb Emt Up	70	0.535315632	1.899532228	0.000201851	0.008959636	0.007507232
Zheng Il22 Signaling Up	23	0.683345709	1.958055934	0.000772125	0.021447923	0.017971101
Wp Notch Signaling Pathway	29	-0.571313694	-1.895322755	0.00100117	0.025478874	0.021348615

GSEA, gene set enrichment analysis.

### 3.6 Gene set variation analysis

**Figure 6:**
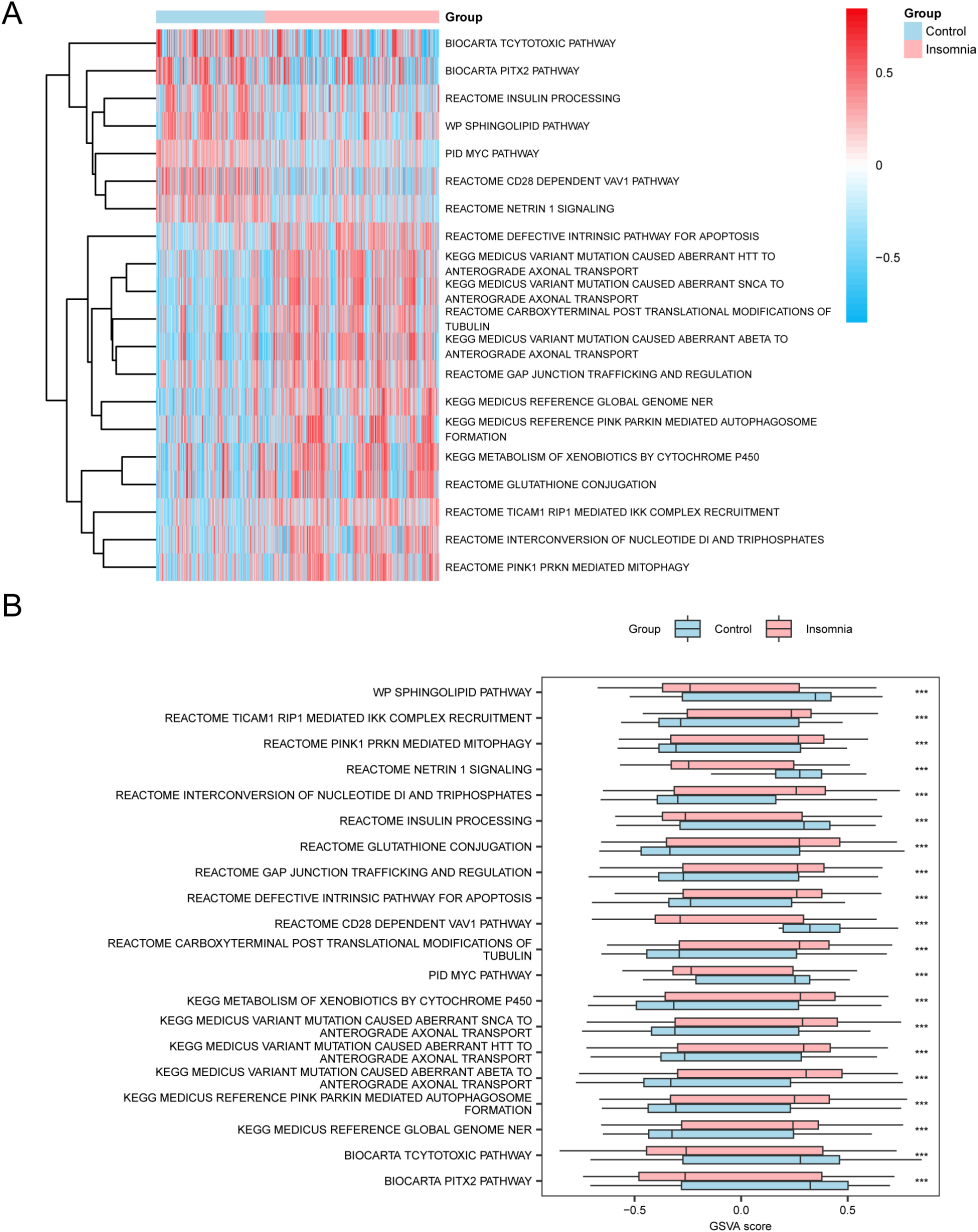
GSVA. **(A, B)** GSVA results in the heat map **(A)** and group comparison diagram **(B)** between the insomnia group and the control group in data set GSE98582. GSVA, gene set variation analysis. *** stands for *p* < 0.001, which is statistically significant. Pink represents the insomnia group, and blue represents the control group. The screening standard of GSVA is *p* < 0.05. In the heat map, blue represents low enrichment and red represents high enrichment.

To explore the differences between c2.cp.v2023.2.Hs.symbols.gmt gene set between the insomnia and control groups in the GSE98582 dataset, GSVA was performed on all genes (**Supplementary Table 11**). The differential expression of the top 20 pathways screened with *p* < 0.05 and in descending order of absolute value of logFC was analyzed and visualized using a heatmap (**Figure 6A**). Differences were verified using the Mann-Whitney U test, and a group comparison chart was drawn (**Figure 6B**). The results of GSVA indicate that the pathways of Reactome Pink1 Prkn-Mediated Mitophagy, Reactome Glutathione Conjugation, Reactome Gap Junction Trafficking and Regulation, Kegg Medicus Variant Mutation Caused Aberrant Abeta to Anterograde Axonal Transport, Kegg Medicus Variant Mutation Caused Aberrant Htt to Anterograde Axonal Transport, and Sphingolipid Pathway were significantly different between the insomnia and control groups (*p* < 0.05).

### 3.7 Protein-protein interaction network

**Figure 7:**
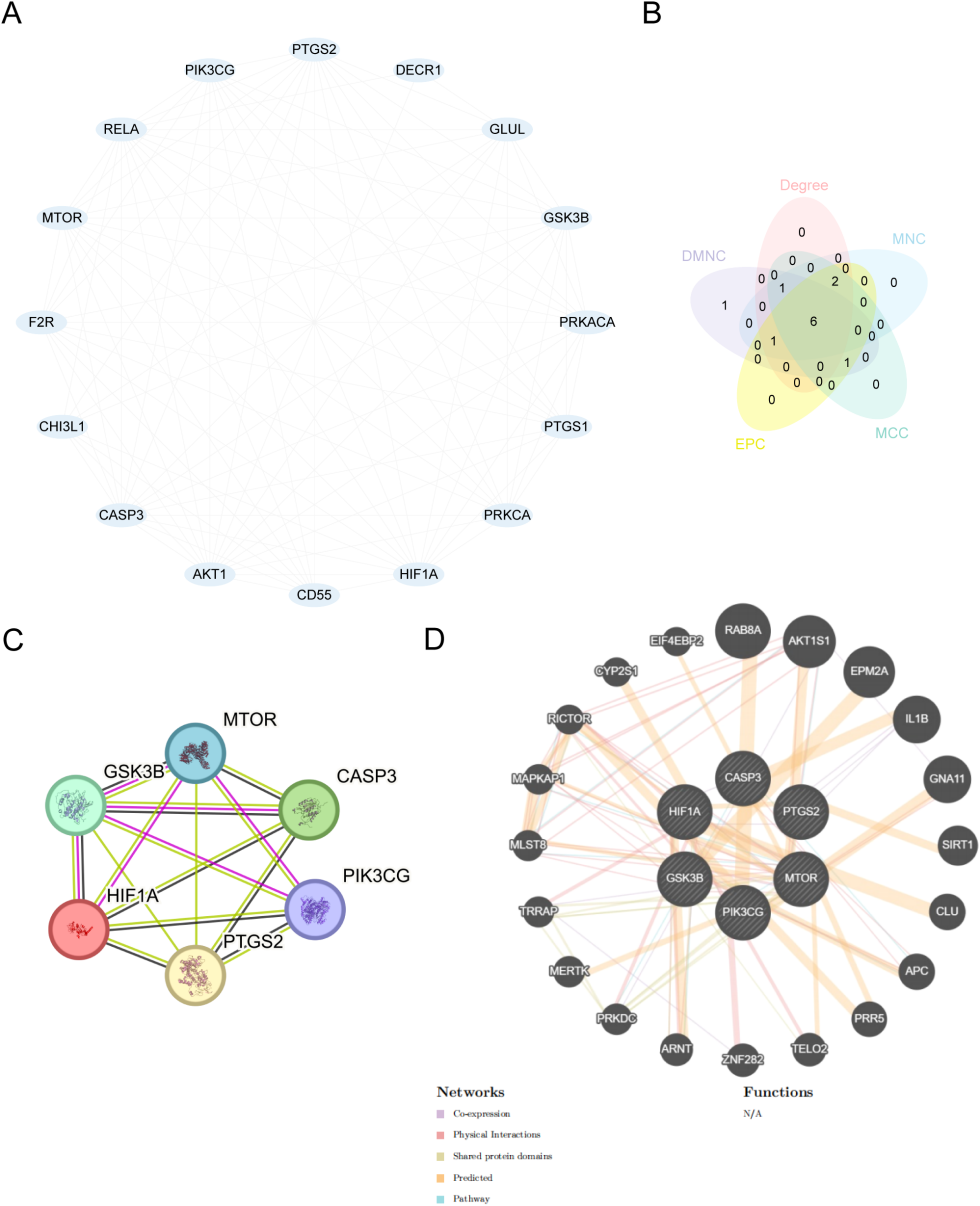
Protein-protein interaction network. **(A)** PPI network of NDTRDEGs. **(B)** Venn diagram of the top six key genes is selected under the five algorithms of MCC, MNC, EPC, degree, and DMNC. **(C)** PPI network of key genes. **(D)** key genes predict the interaction network of genes with similar functions. Lines represent the interaction between genes, and thick lines represent a strong correlation. PPI, protein-protein interaction; degree, degree correlation; MNC, maximum neighborhood component; MCC, maximal clique centrality; EPC, edge percolated component; DMNC, density of maximum neighborhood component.

PPI analysis of the 16 NDTRDEGs was performed using the STRING database (medium confidence, 0.4), and the interaction network was visualized using Cytoscape software (**Figure 7A**). Five algorithms (MCC, MNC, EPC, Degree, and DMNC) were analyzed using the cytoHubba plug-in, and six common genes (HIF1A, PTGS2, CASP3, GSK3B, MTOR, and PIK3CG) in the top six were selected as key genes (**Figures 7B, C**). Similar key genes were predicted using the GeneMANIA website, and an interaction network was drawn to observe the physical interactions between them, sharing protein domains, gene interactions, and other information (**Figure 7D**).

### 3.8 Correlation analysis

**Figure 8:**
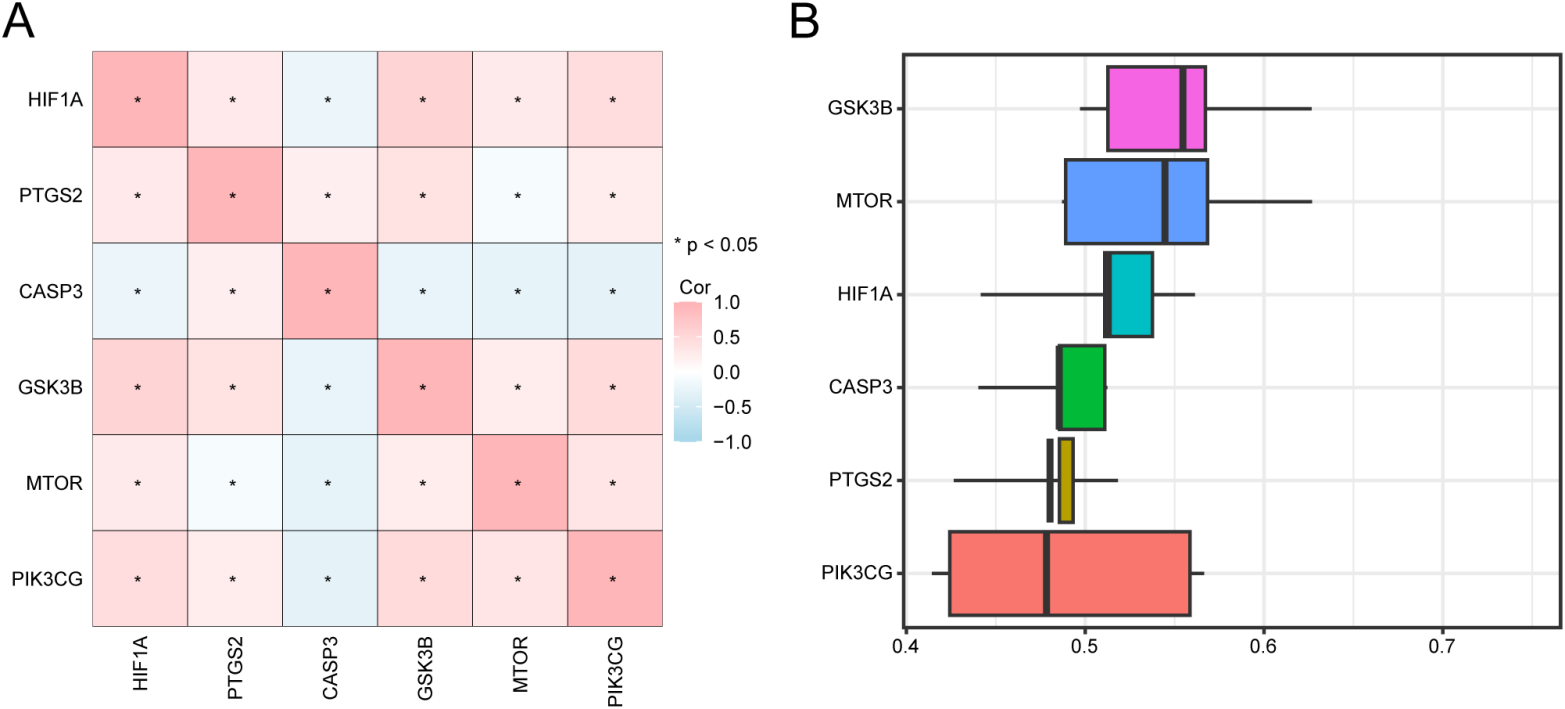
Correlation analysis and interaction analysis. **(A)** Correlation analysis between key genes. **(B)** Functional similarity analysis of key genes. * means *p* < 0.05. The absolute value of the correlation coefficient (r value) indicates weak or uncorrelated below 0.3, weak correlation between 0.3 and 0.5, moderate correlation between 0.5 and 0.8, and strong correlation above 0.8.

Based on the complete expression matrix of the six key genes in the GSE98582 dataset, a correlation heat map was constructed. Our results showed that GSK3B positively correlated with HIF1A and PIK3CG. A negative correlation was noted between CASP3 and MTOR (**Figure 8A**). Functional similarity analysis showed that GSK3B had the highest functional similarity with the other genes (**Figure 8B**).

### 3.9 Polysomnography monitoring

**Figure 9:**
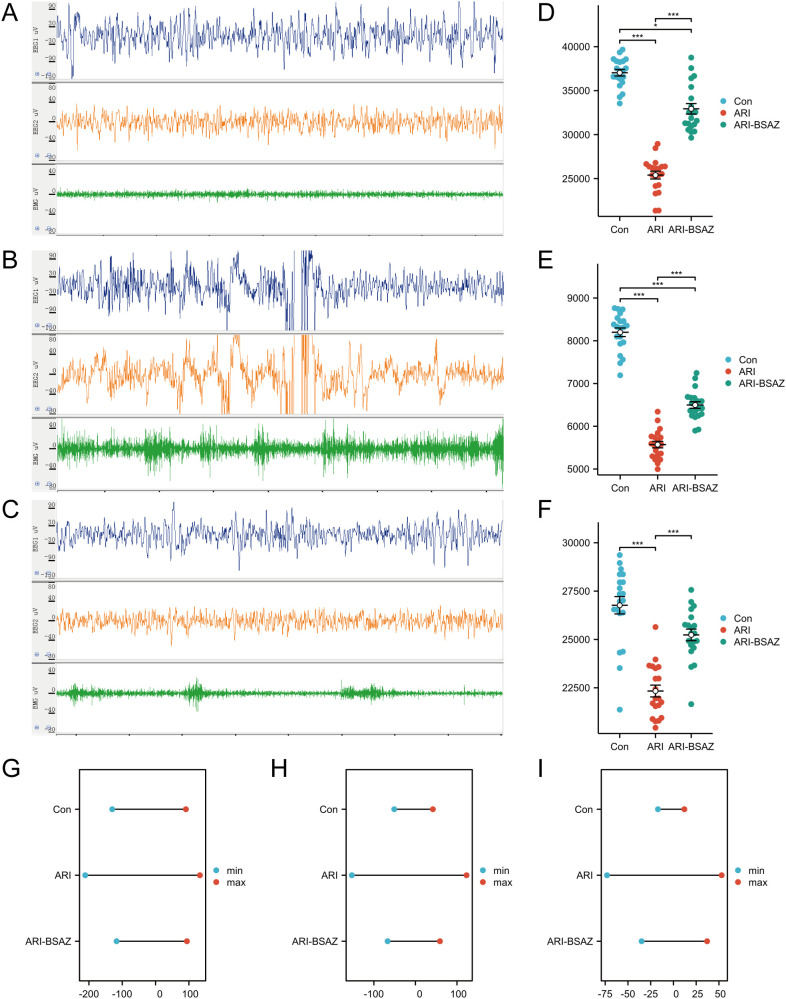
Polysomnography monitors sleep dynamics. **(A)** Polysomnography in the Con group; **(B)** Polysomnography in the ARI group; **(C)** Polysomnography in the ARI-BSAZ group; **(D)** Total sleep time; **(E)** Rapid eye movement sleep time; **(F)** Slow wave sleep time; **(G)** EEG1 voltage; **(H)** EEG2 voltage; **(I)** EMG voltage. Con, control group; ARI, aging-related insomnia model group; ARI-BSAZ, the intervention group of Bushen Anzhi recipe for ARI model. * stands for *p* < 0.05, *** stands for *p* < 0.001.

Polysomnography indicated that the total sleep time in the ARI group (25391.9 ± 1974.4) s was significantly reduced (*p* < 0.001) compared with that in the Con group (37036.8 ± 1668.9) s, and the total sleep time was significantly increased after the intervention with the Bushen Anzhi recipe (32936.0 ± 2668.8) s (*p* < 0.001). The rapid eye movement time in the ARI group (5571.1 ± 340.2) s was significantly decreased compared with that in the Con group (8198.9 ± 449.7) s (*p* < 0.001), and it was significantly increased after the intervention with traditional Chinese medicine (6497.1 ± 344.4) (*p* < 0.001). The slow-wave sleep time showed the same trend. Compared with ARI group (22332.9 ± 1364.8) s, the ARI-BSAZ group (25235.4 ± 1335.8) s had a significantly higher slow-wave sleep time (*p* < 0.001) (**Figures 9A–F**). Similarly, among the three groups of leads, the voltage fluctuation in the ARI group (EEG1: min -211, max 133; EEG2: min-153, max123; EMG: min-73, max53) uV was larger than that in the Con group (EEG1: min-130, max91; EEG2: min-51, max42; EMG: min-17, max12) uV, but the voltage fluctuation decreased after Chinese herbal medicine administration (EEG1: min-117, max94; EEG2: min-67, max59; EMG: min-35, max37) uV, indicating that sleep quality was improved after Chinese herbal medicine intervention (**Figures 9G–I**).

### 3.10 Behavioral assessments and analysis of Wnt pathway-related protein expression

**Figure 10:**
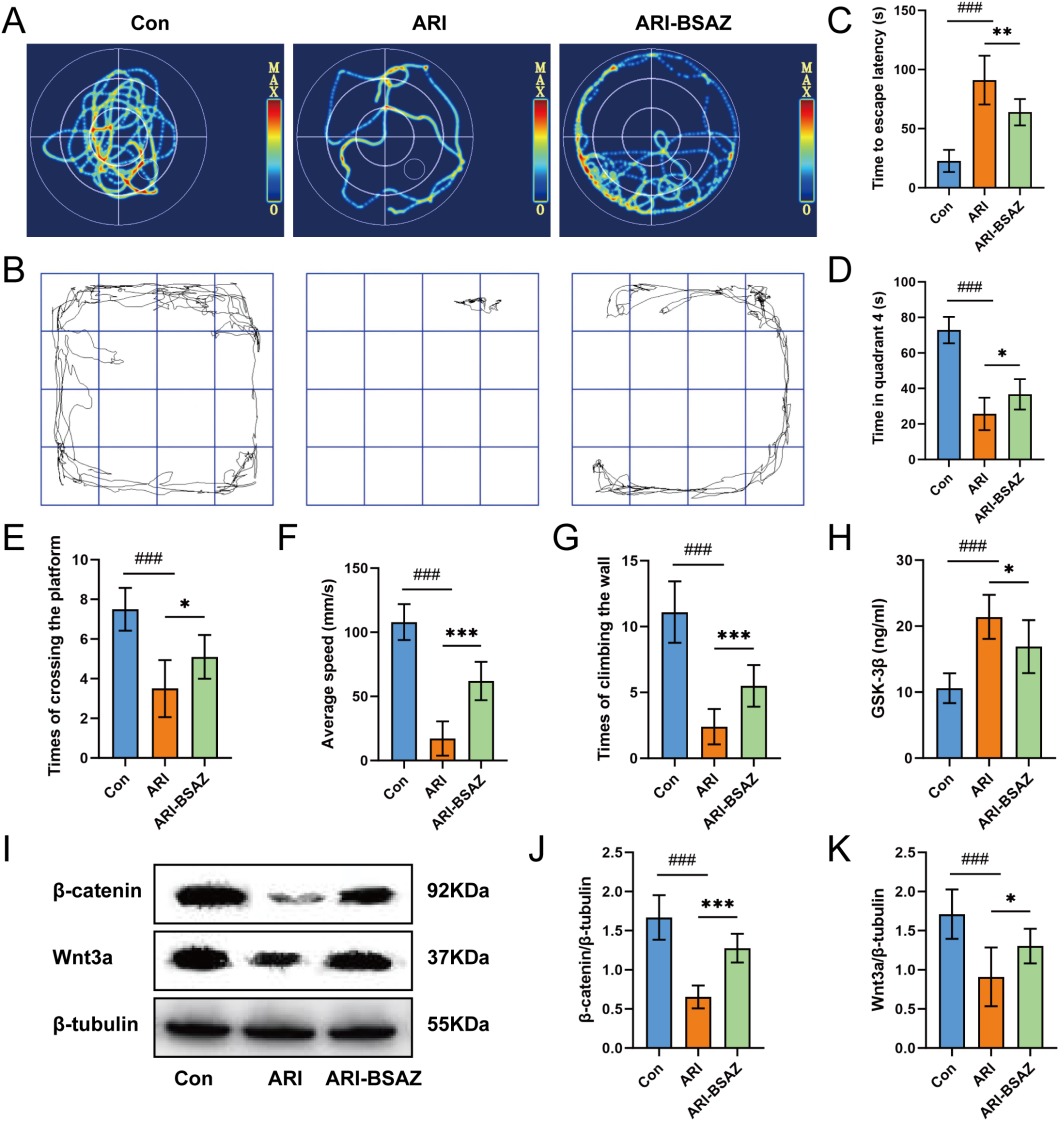
Behavioral assessments and analysis of Wnt pathway-related protein expression. **(A)** Water maze trajectory; **(B)** Open-field trajectory; **(C)** Time to escape latency; **(D)** Time in quadrant 4; **(E)** Times of crossing the platform; **(F)** Average speed; **(G)** Times of climbing a wall; **(H)** GSK-3β content; **(I)** western blot bands of β-catenin and Wnt3a; **(J)** The level of β-catenin; **(K)** The level of Wnt3a. ARI, aging-related insomnia; BSAZ, Bushen Anzhi recipe; ^###^ *p* < 0.001, Con vs. ARI. *p < 0.05, **p < 0.01, ***p < 0.001, ARI vs. ARI-BSAZ.

Morris water maze and open field test were carried out and their tracks were recorded (**Figures 10A, B**). The escape latency time of Con group was significantly shorter than that of ARI group (*p* < 0.001), which indicated that the spatial memory of ARI model group was decreased, and that of ARI-BSAZ group was significantly shorter than ARI group (*p* < 0.01) (**Figure 10C**). At the same time, the stay time in the fourth quadrant of ARI-BSAZ group was significantly longer than ARI group (*p* < 0.05) (**Figure 10D**). The times of crossing the platform in ARI-BSAZ group were more than those in ARI group (*p* < 0.05) (**Figure 10E**). The above results show that BSAZ treatment can significantly improve the spatial memory of ARI rats. In the field experiment, the average speed of the ARI-BSAZ group was significantly higher than that of the ARI group (*p* < 0.001) (**Figure 10F**), and the times of climbing the wall of the ARI-BSAZ group were more than ARI group (*p* < 0.001) (**Figure 10G**), which indicated that the intervention of BSAZ could improve the sports ability and the ability of exploring new things of the ARI rats. The results of ELISA assays revealed that compared with rats in the Con group, there was a significant increase in the levels of GSK-3β in the ARI group rats (*p* < 0.001), and, contrastingly, a reduction in levels in response to the intragastric administration of the BSAZ formulation (*p* < 0.05) (**Figure 10H**). Furthermore, western blotting results indicated that the levels of β-catenin in ARI-BSAZ group rats were significantly higher than those in rats in the ARI group (*p* < 0.001) (**Figure 10J**), as were the levels of Wnt3a (*p* < 0.05) (**Figure 10K**).

## 4 Discussion

Insomnia substantially affects the quality of life of individuals with the condition. Compared with the general population, individuals with nervous system diseases have a higher incidence of insomnia, with the insomnia rate in patients with neurodegenerative diseases ranging from 11% to 74.2%, vascular diseases ranging from 20% to 37%, inflammatory diseases ranging from 13.3% to 50%, and epilepsy ranging from 28.9% to 74.4%. The treatment options include sedatives and antidepressants. Drug therapy is being increasingly questioned during the course of treatment because of potential dependence and drug resistance. Traditional Chinese medicine has attracted increasing global attention as an important component of complementary and integrative health approaches (29). However, the therapeutic targets through which Chinese herbal medicines exert their effects against insomnia remain unclear. This study comprehensively analyzed the relationship between the active components of Chinese herbal medicines and insomnia-related genes by integrating various bioinformatics methods, providing a theoretical basis for developing new drugs. In the present study, 4,173 DEGs were identified from the GSE98582 dataset, of which 1,505 were upregulated, and 2,668 were downregulated. Sixteen NDTRDEGs were identified as key genes, including PTGS1, CD55, PRKCA, and HIF1A. These key genes may be involved in the process of insomnia, providing clues for understanding the mechanism of insomnia. In the process of screening DEGs, we adopted a relatively relaxed fold-change threshold, considered sufficient for exploratory analysis, aiming at capturing the potential biological signals associated with insomnia and neuroinflammation as comprehensively as possible. A relatively relaxed fold change threshold is used to improve the detection sensitivity, but it may reduce the specificity, so the interpretation of the results is mainly based on the robustness of the pathway and network level, rather than the significance of a single gene. Given that a certain degree of statistical noise may be introduced at the initial screening stage, the candidate genes were gradually converged and constrained via multiple intersection screening of neuroinflammation-related gene sets and Chinese medicine target genes, protein interaction network construction and multi-level functional enrichment analysis, thereby contributing to an enhancement of the biological specificity and overall reliability of the results.

Previous studies have shown that quercetin and atropine, core components of *Ziziphus jujuba* Mill., possess binding affinity to PTGS1, indicating that PTGS1 may be involved in biological pathways related to sedation and neuroactive ligand-receptor interactions (30). CD55 produced by neuronal cells attaches to fibers around the nerve plexus, where it may contribute to protection of glial cells during complement-mediated injury (31). However, CD55 has different functions in different cell groups, and the level of CD55 expression in peripheral immune cells of patients with schizophrenia is significantly higher than that in healthy individuals (32). Through molecular docking analysis, the main component β-sitosterol in *Pinellia ternata* and Coix seed decoction exhibits strong binding affinity for PRKCA, and may improve insomnia by regulating the serotonin pathway (33). PRKCA is involved in a variety of signaling pathways (such as neurotransmitter release and synaptic plasticity), which may affect sleep by regulating GABA and glutamate (34). The circadian rhythm gene HIF1A is regulated by four genes, Rel, Polr2A, Mafk, and Srbf1, and plays a key role in circadian regulation and influences pathways related to melatonin synthesis (35). HIF1A is the core transcription factor required for cells to cope with hypoxia and regulate energy metabolism and angiogenesis. The HIF1A pathway may improve sleep disorders by regulating oxygen homeostasis and metabolism (36).

In the present study, all genes in the GSE98582 dataset were significantly enriched in the Wnt, Notch, and other signaling pathways. The results of GSVA showed that the Reactome Pink1 Prkn-Mediated Mitophagy, Reactome Glutathione Conjugation, and Sphingolipid Pathways were significantly different in the DEG analysis, which may reveal the important role of NDTRDEGs in cell signal transduction and immune response in insomnia. Treatment with Suanzaoren Decoction significantly reversed the depression-like behavior of rats, increased BDNF expression, inhibited the activation of the Wnt/β-catenin pathway, and reduced the nuclear translocation of NF-κB and β-catenin (37). Genome-wide association studies were performed on 113,006 individuals, and the correlation detection results of insomnia enhancement subregions showed that the Notch Signaling Pathway was related to insomnia, and insomnia-related genes were significantly enriched in neural stem cells (38). Our previous study showed that GAD67 has a negative regulatory effect on insomnia and that polygalanin can regulate insomnia by mediating the expression of cytokines in the Keap1/Nrf2/Parkin/PINK1 pathway regulated by GAD67 (39). Glutathione (GSH) is the primary intracellular antioxidant, and decreased GSH levels may lead to increase in oxidative stress, interfere with the balance of neurotransmitters (such as GABA and melatonin), and affect the sleep-wake cycle (40). In the future, the treatment of different types of insomnia can be guided by analyzing the GSH levels in patients with insomnia.

Construction of a PPI network helped identify six key target genes, HIF1A, PTGS2, CASP3, GSK3B, MTOR, and PIK3CG, among them, HIF1A, PTGS2, CASP3 and GSK3B were significantly up-regulated in insomnia group, while MTOR and PIK3CG were down-regulated (*p* < 0.05), which can be used as potential therapeutic targets. It should be emphasized that the PPI network constructed in this study and hub gene screening based on cytoHubba were primarily employed to identify candidate genes that may have a key regulatory significance from the perspective of network topology, and to provide reference for multi-level mechanism integration and biological interpretation, rather than to provide direct evidence for determining protein interactions or causality. Similar analysis strategies have been adopted in recent studies. Chen et al. obtained the complete transcriptome sequencing data of Major depressive disorder from three different brain regions from GEO database, used R-package “limma” to screen DEGs, used STRING to construct PPI network, and visualized it in Cytoscape. 342 DEGs were selected to be related to amygdala, 76 DEGs to anterior cingulate cortex and 64 DEGs to dorsolateral prefrontal cortex (p < 0.05, |logFC| > 0.15) (41). Thirty-eight compounds in valerian volatile oil were predicted to affect 103 insomnia-related targets, including PTGS2, dopamine receptor D2, and serotonin receptor 2A, which are helpful in regulating neuroactive ligand-receptor interactions and significantly reducing sleep latency (42). *Ziziphus jujuba* seeds may promote sleep, through strong binding affinity for PTGS2 and PTGS1, through active compounds such as quercetin (43). In this study, the functional similarity between GSK3B and other key genes was the highest and was predicted to have a strong correlation with insomnia. Studies have shown that GSK3B is associated with insomnia in patients with depression and that targeted therapy for patients with severe insomnia may be effective (44). In the animal experiments performed in the present study, we observed alterations consistent with dysregulation of the Wnt signaling pathway, including reduced β-catenin protein levels and elevated GSK-3β activity. Following administration of the Bushen Anzhi formulation, a reversal in the levels of these proteins was detected, suggesting a potential restoration of Wnt pathway activity, which may contribute to the attenuation of nerve cell apoptosis and synaptic dysfunction, though direct evidence for these downstream effects requires further investigation. These findings provide preliminary evidence for the potential involvement of GSK-3β, β-catenin, and Wnt3a in the sleep-regulatory effects of this formulation, and corroborate the transcriptomic predictions at the molecular and protein levels.

Electroencephalography is a noninvasive technique that is widely used to objectively evaluate sleep parameters, such as sleep latency and awakening times, in patients with insomnia. These patients show increased β wave power, increased θ and γ energy during waking, and increased α and σ energy during rapid eye movement (REM) sleep (45). A clinical study of 48 patients with primary insomnia and 30 patients with good sleep found that the δ wave power of patients with insomnia was significantly decreased, while the β1, β2, and γ wave powers were significantly increased (46). This is consistent with the findings of this study: slow-wave sleep (σ wave) is significantly reduced in insomnia model rats, and it is significantly increased after gastric administration of Chinese herbal medicine, indicating that Bushen Anzhi recipe may improve sleep quality in older adults with insomnia.

Nevertheless, this study has some limitations. First, 342 samples from patients with insomnia and 213 control samples were analyzed, the sample size was relatively small. Insomnia is a heterogeneous condition, and, accordingly, verification among multiple datasets can contribute to consolidating the overall findings. The bioinformatics analysis conducted in this study was based primarily on reference to a single public database. In this regard, differences in sample sources, population characteristics and detection platforms among different research cohorts may have a certain influence on the results obtained for gene expression patterns and pathway enrichment, thus limiting a generalization of the relevant conclusions to a wider population. In the future, independent queue data from different sources will be integrated on this basis for further verification and expansion. In addition, PPI network is constructed with a lower confidence threshold to obtain more comprehensive interaction information, but this setting may introduce some weakly supported connections. The determination of key genes is mainly based on the stable results obtained by multi-algorithm cross-screening, so the relevant explanation should be understood as an exploratory conclusion, rather than a conclusive judgment of topological centrality under a specific threshold. Second, although EEG monitoring shows a high value in insomnia, it is still challenging due to the lack of standardization and large individual differences. Future research should focus on multimodal integration, such as combining fMRI and genetic data, to deepen our understanding of the neural mechanisms of insomnia and develop targeted interventions. Finally, this study employed a D-galactose combined with PCPA-induced aging insomnia model, which primarily simulates aging-associated oxidative stress and serotonergic system dysfunction, and can stably reproduce alterations in sleep architecture for evaluating the effects of interventions under specific neurobiochemical conditions. However, elderly insomnia in humans involves multiple regulatory mechanisms, including psychological, circadian, metabolic, and neuroimmune factors, which the current model cannot fully capture in terms of its complex pathological processes. Therefore, the findings of this study mainly reflect the sleep-regulatory effects of the Bushen Anzhi formula under specific pathological conditions, and further research is needed to support extrapolation of these results to the overall clinical course of elderly insomnia in humans.

In summary, this study revealed, for the first time, that the Bushen Anzhi recipe may exert anti-insomnia effects through multiple targets and pathways. Its core mechanism may involve the regulation of key genes such as HIF1A, PTGS2, CASP3, and GSK3B, and that its bioactive constituents may modulate the Wnt signaling pathway, thereby restoring immune homeostasis and inducing functional alterations in cellular processes. It should be noted that the relationship between the observed therapeutic effects of the Bushen Anzhi formulation and Wnt signaling activity identified in the present study is associative in nature. Future studies employing pathway-specific perturbation strategies — such as pharmacological inhibition of GSK-3β, siRNA-mediated knockdown of β-catenin, or Wnt pathway rescue assays — will be necessary to formally establish a causal mechanistic role. Furthermore, the compositional complexity of the formula presents a significant challenge, as the individual contributions of each constituent to the identified targets, as well as the intricate interaction networks among these components, remain to be fully elucidated. In the future, the promotion mechanism of slow-wave sleep should be clarified using electrophysiology and molecular biology. Further exploration of the potential of this prescription for insomnia or neurodegenerative diseases will provide a scientific basis for developing a Chinese herbal medicine treatment for insomnia.

## Data Availability

The original contributions presented in the study are included in the article/**Supplementary Material**. Further inquiries can be directed to the corresponding author.
