# Supplementary figures and images for: Bioinformatics analysis of the mechanisms and efficacy of the Bushen Anzhi recipe in treating aging-related insomnia
Source: Front Psychiatry. 2026 May 8;17:1770410. doi: 10.3389/fpsyt.2026.1770410 (PMC13194403; doi:10.3389/fpsyt.2026.1770410)

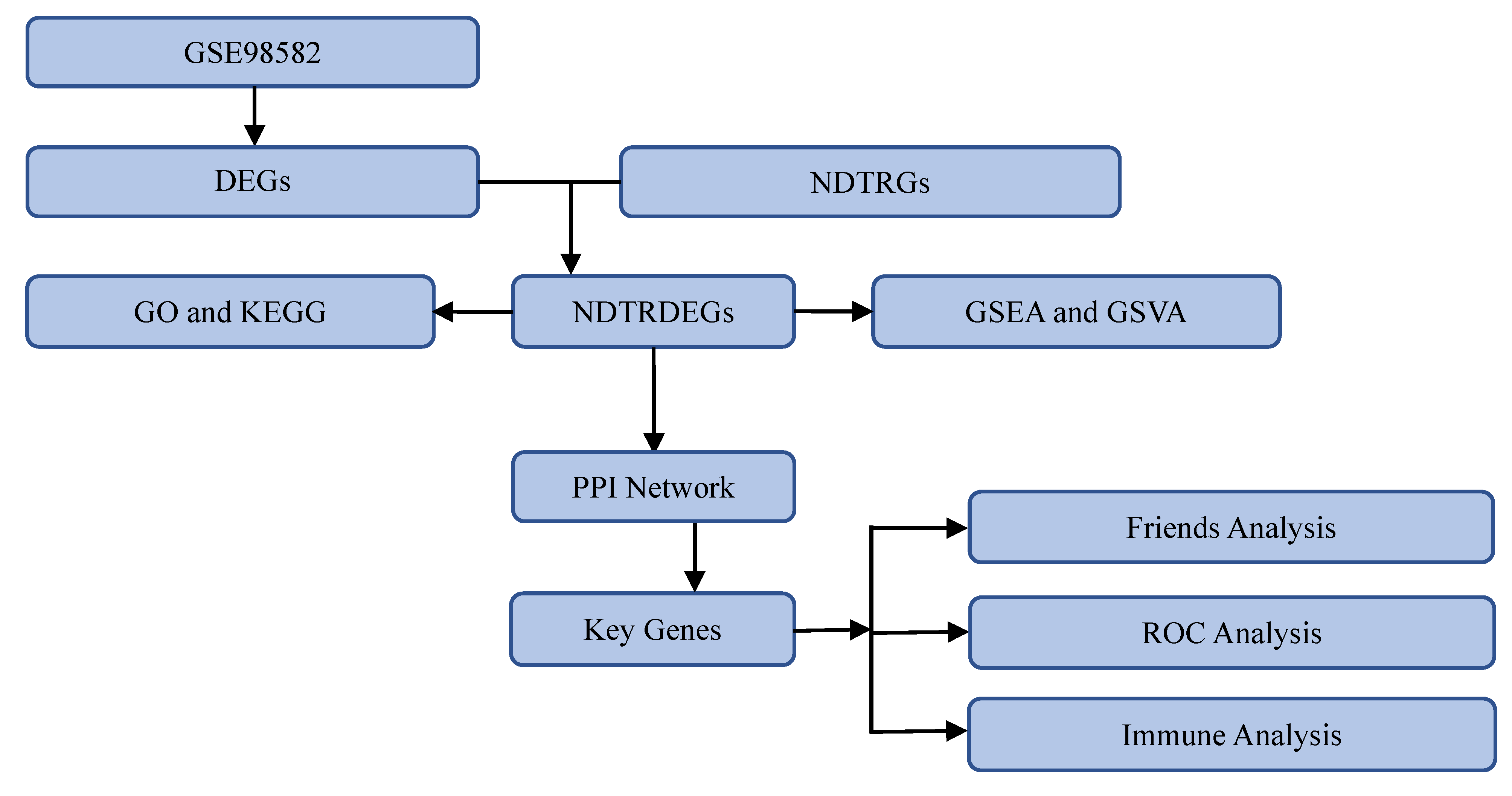

Supplement: Supplementary file 1 [file Image1.tif]

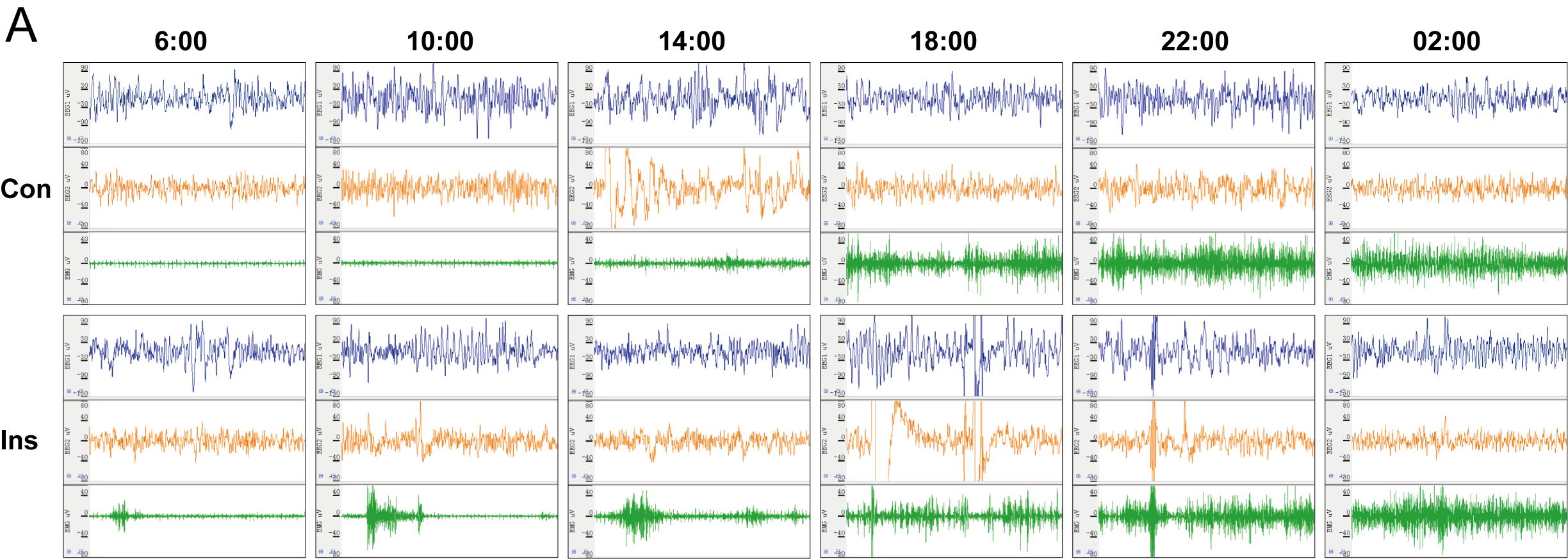

Supplement: Supplementary file 2 [file Image2.tif]

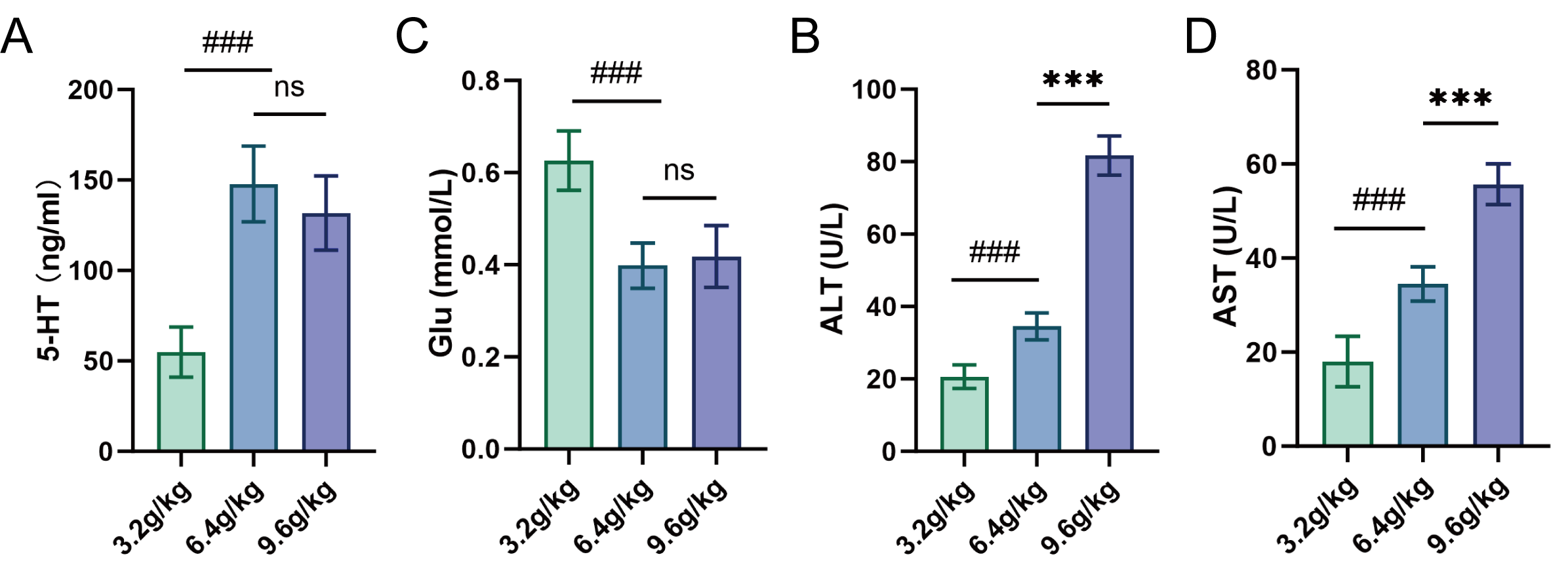

Supplement: Supplementary file 3 [file Image3.tif]

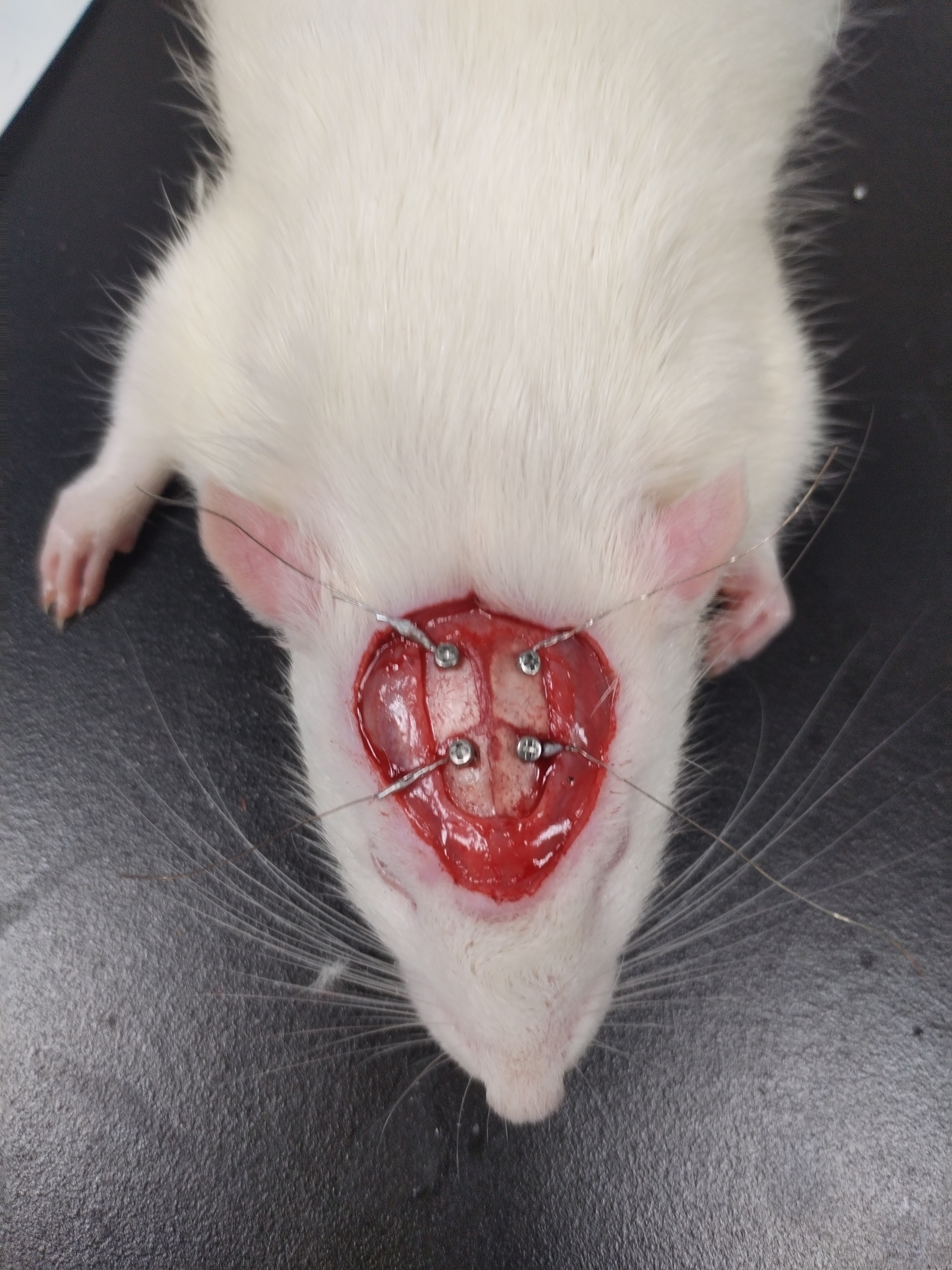

Supplement: Supplementary file 4 [file Image4.tif]
