# Supplementary material for: Bioinformatics analysis of the mechanisms and efficacy of the Bushen Anzhi recipe in treating aging-related insomnia
Source: Front Psychiatry. 2026 May 8;17:1770410. doi: 10.3389/fpsyt.2026.1770410 (PMC13194403; doi:10.3389/fpsyt.2026.1770410)
Supplement: Supplementary file 11 [file Table4.doc]

Table S12. Statistical method

| Analysis item | Statistical test method | Multiple-testing correction method | Statistical unit |
| --- | --- | --- | --- |
| DEG | Limma (Bayesian method based on linear model) | unadjusted (*p* < 0.05) | Gene |
| GO/KEGG | Hypergeometric distribution test (clusterProfiler package) | FDR (Benjamini-Hochberg) adjusted (*q* < 0.05) | Gene set (GO item/KEGG pathway) |
| GSEA | Permutation test (to evaluate the significance of enrichment fraction) | FDR adjusted (*q* < 0.25) | Gene set in MSigDB database |
| GSVA | Limma (Analyze the differences between groups of GSVA score matrix) | FDR adjusted (*q* < 0.25) | Gene sets (pathways in MSigDB database) |
| ssGSEA | Calculate the enrichment fraction of immune cells, Wilcoxon was used for comparison between groups. | Original p value (*p* < 0.05), Benjamini-Hochberg (Figure A) | Immune cell type |
| Correlation analysis | Spearman test | Unadjusted (*p* < 0.05) | Gene pair/gene immune cell pair |
