# Supplementary material for: Bioinformatics analysis of the mechanisms and efficacy of the Bushen Anzhi recipe in treating aging-related insomnia
Source: Front Psychiatry. 2026 May 8;17:1770410. doi: 10.3389/fpsyt.2026.1770410 (PMC13194403; doi:10.3389/fpsyt.2026.1770410)
Supplement: Supplementary file 15 [file Table8.docx]

### Table 4. Results of GSVA for Combined Datasets.

| Pathway | logFC | AveExpr | t | P.Value | adj.P.Val | B |
| --- | --- | --- | --- | --- | --- | --- |
| REACTOME CD28 DEPENDENT VAV1 PATHWAY | -0.443816333 | 0.016673118 | -9.66433123 | 1.45E-20 | 2.72E-17 | 35.92827774 |
| KEGG MEDICUS VARIANT MUTATION CAUSED ABERRANT ABETA TO ANTEROGRADE AXONAL TRANSPORT | 0.434612619 | 0.002838814 | 8.371649391 | 4.40E-16 | 1.65E-13 | 25.88980373 |
| KEGG MEDICUS VARIANT MUTATION CAUSED ABERRANT SNCA TO ANTEROGRADE AXONAL TRANSPORT | 0.372549939 | 0.013263482 | 7.553321543 | 1.69E-13 | 1.87E-11 | 20.11574999 |
| REACTOME CARBOXYTERMINAL POST TRANSLATIONAL MODIFICATIONS OF TUBULIN | 0.358809129 | 0.006899263 | 7.611100386 | 1.13E-13 | 1.33E-11 | 20.50776528 |
| REACTOME NETRIN 1 SIGNALING | -0.358779051 | 0.016496184 | -9.388304703 | 1.43E-19 | 1.35E-16 | 33.69558384 |
| KEGG MEDICUS REFERENCE GLOBAL GENOME NER | 0.35534996 | -0.008714408 | 7.649867524 | 8.59E-14 | 1.12E-11 | 20.77215153 |
| REACTOME DEFECTIVE INTRINSIC PATHWAY FOR APOPTOSIS | 0.35342462 | 0.015262995 | 8.340689136 | 5.55E-16 | 1.74E-13 | 25.66283376 |
| KEGG METABOLISM OF XENOBIOTICS BY CYTOCHROME P450 | 0.347983358 | 0.001973892 | 6.542695851 | 1.34E-10 | 6.48E-09 | 13.6628215 |
| KEGG MEDICUS VARIANT MUTATION CAUSED ABERRANT HTT TO ANTEROGRADE AXONAL TRANSPORT | 0.3424684 | 0.029038716 | 7.213146236 | 1.75E-12 | 1.56E-10 | 17.85752787 |
| KEGG MEDICUS REFERENCE PINK PARKIN MEDIATED AUTOPHAGOSOME FORMATION | 0.341864861 | -0.008938684 | 6.877910683 | 1.60E-11 | 1.20E-09 | 15.71694071 |
| WP SPHINGOLIPID PATHWAY | -0.324878316 | 0.017015993 | -7.240535387 | 1.45E-12 | 1.36E-10 | 18.0361674 |
| REACTOME GAP JUNCTION TRAFFICKING AND REGULATION | 0.323618991 | 0.025282115 | 7.036168709 | 5.68E-12 | 4.64E-10 | 16.71683794 |
| BIOCARTA PITX2 PATHWAY | -0.322386564 | 0.014922714 | -5.663857934 | 2.35E-08 | 4.55E-07 | 8.702284903 |
| REACTOME INTERCONVERSION OF NUCLEOTIDE DI AND TRIPHOSPHATES | 0.321982773 | -0.024264137 | 6.82723739 | 2.22E-11 | 1.49E-09 | 15.4008362 |
| REACTOME PINK1 PRKN MEDIATED MITOPHAGY | 0.318076972 | 0.004886931 | 6.866069444 | 1.73E-11 | 1.25E-09 | 15.64289709 |
| REACTOME GLUTATHIONE CONJUGATION | 0.31438524 | -0.010018033 | 5.917482637 | 5.64E-09 | 1.45E-07 | 10.06938448 |
| PID MYC PATHWAY | -0.310293092 | 0.001494962 | -8.420650033 | 3.03E-16 | 1.43E-13 | 26.25036334 |
| REACTOME TICAM1 RIP1 MEDIATED IKK COMPLEX RECRUITMENT | 0.306940823 | 0.01092059 | 7.660821942 | 7.96E-14 | 1.12E-11 | 20.84705653 |
| REACTOME INSULIN PROCESSING | -0.306249599 | -0.001025815 | -6.697065493 | 5.10E-11 | 3.09E-09 | 14.59790616 |
| BIOCARTA TCYTOTOXIC PATHWAY | -0.305569589 | 0.000645443 | -5.380492996 | 1.09E-07 | 1.69E-06 | 7.238236762 |
